# Modelling Radiation-Induced Salivary Dysfunction during IMRT and Chemotherapy for Nasopharyngeal Cancer Patients

**DOI:** 10.3390/cancers13163983

**Published:** 2021-08-06

**Authors:** Anna Cavallo, Nicola Alessandro Iacovelli, Nadia Facchinetti, Tiziana Rancati, Salvatore Alfieri, Tommaso Giandini, Alessandro Cicchetti, Carlo Fallai, Rossana Ingargiola, Lisa Licitra, Laura Locati, Stefano Cavalieri, Emanuele Pignoli, Domenico Attilio Romanello, Riccardo Valdagni, Ester Orlandi

**Affiliations:** 1 Department of Medical Physics, Fondazione IRCCS Istituto Nazionale dei Tumori, 20133 Milan, Italy; anna.cavallo@istitutotumori.mi.it (A.C.); Tommaso.giandini@istitutotumori.mi.it (T.G.); emanuele.pignoli@istitutotumori.mi.it (E.P.); 2 Department of Radiation Oncology 2, Fondazione IRCCS Istituto Nazionale dei Tumori, 20133 Milan, Italy; nicolaalessandro.iacovelli@istitutotumori.mi.it (N.A.I.); nadia.facchinetti@cnao.it (N.F.); Carlo.fallai@istitutotumori.mi.it (C.F.); rossana.ingargiola@cnao.it (R.I.); Domenico.Romanello@sabes.it (D.A.R.); ester.orlandi@cnao.it (E.O.); 3 National Center for Oncological Hadrontherapy (CNAO), Clinical Trial Center, 27100 Pavia, Italy; 4 Prostate Cancer Program, Fondazione IRCCS Istituto Nazionale dei Tumori, 20133 Milan, Italy; alessandro.cicchetti@istitutotumori.mi.it (A.C.); Riccardo.valdagni@istitutotumori.mi.it (R.V.); 5 Department of Medical Oncology 3, Fondazione IRCCS Istituto Nazionale dei Tumori, 20133 Milan, Italy; salvatore.alfieri@cro.it (S.A.); lisa.licitra@istitutotumori.mi.it (L.L.); laura.locati@istitutotumori.mi.it (L.L.); stefano.cavalieri@istitutotumori.mi.it (S.C.); 6 Centro di Riferimento Oncologico di Aviano (PN) CRO IRCCS, Department of Medical Oncology, 33018 Aviano, Italy; 7 National Center for Oncological Hadrontherapy (CNAO), Radiation Oncology Clinical Department, 27100 Pavia, Italy; 8 Department of Oncolgy and Hemato-Oncology, Università Degli Studi di Milano, 20122 Milan, Italy; 9 Department of Radiation Oncology 1, Fondazione IRCCS Istituto Nazionale dei Tumori, 20133 Milan, Italy

**Keywords:** nasopharyngeal cancer, radiotherapy, acute toxicity, NTCP modelling, validation

## Abstract

**Simple Summary:**

We built a predictive model for acute salivary dysfunction for nasopharyngeal cancer patients receiving combined treatment. The final aim was to provide a nomogram (with dosimetric and clinical risk factors) to help physicians in the streamline prevention and management of this acute side effect. No research has focused on predicting acute xerostomia so far. We do not know if models predicting late xerostomia can also be applied to acute xerostomia, since different pathogenesis is suggested for acute and late events. The model was tested in two independent external cohorts. Validation results highlighted that the dosimetric part of the predictive model was highly generalisable, with the clinical risk part still being a weak component. The good validation of the model’s discriminative power and of the effect of the size of dosimetric factors created confidence for considering these factors while optimising radiotherapy.

**Abstract:**

Background: Radiation-induced xerostomia is one of the most prevalent adverse effects of head and neck cancer treatment, and it could seriously affect patients’ qualities of life. It results primarily from damage to the salivary glands, but its onset and severity may also be influenced by other patient-, tumour-, and treatment-related factors. We aimed to build and validate a predictive model for acute salivary dysfunction (aSD) for locally advanced nasopharyngeal carcinoma (NPC) patients by combining clinical and dosimetric factors. Methods: A cohort of consecutive NPC patients treated curatively with IMRT and chemotherapy at 70 Gy (2–2.12 Gy/fraction) were utilised. Parotid glands (cPG, considered as a single organ) and the oral cavity (OC) were selected as organs-at-risk. The aSD was assessed at baseline and weekly during RT, grade ≥ 2 aSD chosen as the endpoint. Dose-volume histograms were reduced to the Equivalent Uniform Dose (EUD). Dosimetric and clinical/treatment features selected via LASSO were inserted into a multivariable logistic model. Model validation was performed on two cohorts of patients with prospective aSD, and scored using the same schedule/scale: a cohort (NPC_V) of NPC patients (as in model training), and a cohort of mixed non-NPC head and neck cancer patients (HNC_V). Results: The model training cohort included 132 patients. Grade ≥ 2 aSD was reported in 90 patients (68.2%). Analyses resulted in a 4-variables model, including doses of up to 98% of cPG (cPG_D98%, OR = 1.04), EUD to OC with *n* = 0.05 (OR = 1.11), age (OR = 1.08, 5-year interval) and smoking history (OR = 1.37, yes vs. no). Calibration was good. The NPC_V cohort included 38 patients, with aSD scored in 34 patients (89.5%); the HNC_V cohort included 93 patients, 77 with aSD (92.8%). As a general observation, the incidence of aSD was significantly different in the training and validation populations (*p* = 0.01), thus impairing calibration-in-the-large. At the same time, the effect size for the two dosimetric factors was confirmed. Discrimination was also satisfactory in both cohorts: AUC was 0.73, and 0.68 in NPC_V and HNC_V cohorts, respectively. Conclusion: cPG D98% and the high doses received by small OC volumes were found to have the most impact on grade ≥ 2 acute xerostomia, with age and smoking history acting as a dose-modifying factor. Findings on the development population were confirmed in two prospectively collected validation populations.

## 1. Introduction

Xerostomia, or oral dryness, is one of the most prevalent and challenging adverse effects of radiation therapy (RT) among locally advanced head and neck cancer (HNC) patients, and this is particularly true in the context of nasopharyngeal cancer (NPC) treatments, even in the intensity-modulated RT (IMRT) era [1,2,3,4]. Xerostomia represents toxicity that could resolve over time [4,5,6,7]. Still, it often translates into a permanent condition that seriously affects swallowing, speaking and oral health, impairing several domains of patients’ qualities of life (QoL) [1,8].

Although radiation-induced xerostomia is multifactorial, it primarily results from damage to the major salivary glands that are usually included in the radiation fields. Parotid glands are responsible for approximately 90% of salivary output [9,10]: radiation results in a decrease in salivary flow and a change in salivary composition, leading to a sense of dry mouth and sticky saliva.

Salivary glands are especially sensitive to radiation, exhibiting both acute (within 90 days of the RT treatment completion) and chronic (after 90 days from the end of treatment) responses to radiotherapy [11]. It is postulated that different pathogeneses of acute and late salivary alterations exist: early response results in the atrophy of the salivary gland acinar cells show that glandular shrinkage without inflammation might be due to radiation-induced apoptosis, whereas late response with inflammation appears as an attrition of the acinar cells followed by the replacement with fibrotic tissue, which could be a result of radiation-induced necrosis [11,12]. In addition, RT-induced mechanisms of salivary gland dysfunction which also include the effect of irradiation on adjacent non-irradiated tissue via paracrine, autocrine and direct cell–to-cell interactions, coined the bystander effect in other models of RT-induced damage [13].

Due to the negative impact of late salivary dysfunction on HNC patients’ QoL and the lack of an effective management strategy [14], a great deal of effort has been made in the last few decades to identify patient- and therapy-related factors and to build predictive models for late toxicity to determine treatment planning optimisation goals to possibly minimise the insurgence of such side effects, i.e., identify organs to be possibly spared and dose volume cutoffs to be utilised [15,16,17,18,19,20].

However, salivary alterations also occur during treatment as one of the most frequent acute toxicities leading to oral discomfort, taste alteration and the impairment of chewing and swallowing functions, which can bring about nutritional depletion, weight loss and, in some cases, even treatment interruptions [1]. In addition, acute xerostomia during weeks 3–6 of RT is a significant prognostic factor for severe late dysphagia in a large HNC population [21]. This raises the hypothesis that the radiation dose causes both acute and late toxicity; the acute toxicity alone sets in motion a series of effects that ultimately lead to late toxicity. This would imply that acute toxicities are at least partly in the causal pathway from radiation dose to late toxicity. Such causality cannot be proven from observational data and should therefore be studied and established in a biological, experimental setting.

To date, little research has focused on the prediction of acute xerostomia during RT treatment, and we do not know if models predicting late xerostomia can be correctly applied to acute toxicity since different pathogeneses are suggested for acute and late events.

This study aimed to build a predictive model for acute salivary dysfunction during curative IMRT for locally advanced NPC patients with chemotherapy treatment by considering clinical, treatment and dosimetric parameters. Eventually, a nomogram was derived from the model so that physicians could better prevent and manage this acute side effect.

## 2. Materials and Methods

### 2.1. Patient Population

For model development, a series of consecutive non-metastatic NPC patients were considered. All patients were curatively treated with IMRT with chemotherapy between 2004 and 2015. The inclusion criteria were the availability of a detailed anamnesis, dosimetric data (in terms of DICOM-RT) and weekly assessments of acute toxicities. Patients with any symptoms of acute salivary dysfunction or oral mucositis prior to RT initiation were excluded. Eligible patients were identified from an institutional clinical database. This retrospective study received the approval of the internal ethics committee.

Validation was performed with two cohorts of patients, treated at the same department from 2017–2018, within a prospective trial devoted to modelling radio-induced toxicity approved by the internal ethics committee. The first cohort (NPC_V) consisted of NPC patients (as in the model training phase), while the second cohort was a mixed population of non-NPC HNC patients (HNC_V). In these two cohorts, acute toxicity was scored prospectively, using the same schedule and scale as those used in the NPC cohort used to train the model.

### 2.2. Treatment: Delineation of Organs at Risk, Constraint Planning and Selection of Toxicity-Related Organs at Risk

Planning target volumes (PTVs), IMRT planning and delivery characteristics were formerly described [22,23]. Regarding the delineation of organs at risk (OARs), parotid glands (PGs) and the oral cavity (OC) were contoured according to van de Water et al. [24] and Eisbruch et al. [25], respectively. The OC included the surface of the inner lips, buccal mucosa, tongue, base of the tongue, the floor of the mouth and palate. The planning dose objectives were as follows: mean dose ≤ 26 Gy and/or volume receiving 30 Gy (V30 Gy) ≤ 50% for at least one PG and mean dose ≤ 40 Gy for OC. As most NPC patients showed a primary tumour including the entire nasopharynx and/or massive retropharyngeal bilateral node involvement and/or N2-N3 disease, it was not feasible to distinguish between ipsilateral and contralateral PGs. Therefore, for this study, the PGs were considered together as the sum of the volume of both glands, thus originating a single OAR: ‘combined PGs’ (cPG). Any OC components in superposition with gross target volumes (GTVs) were excluded from the OC contour. Lower priority was given to PGs and OC constraints during treatment planning after sparing neurological structures and ensuring target coverage.

Both cPG and OC were considered for acute salivary dysfunction modelling, as cPGs were responsible for most of the salivary flow. At the same time, the OC acted as a surrogate of minor salivary glands. Conversely, submandibular glands were not taken into account since they were entirely included in the PTVs and thus, were always receiving a mean dose similar to the PTV prescription (i.e., ≥50 Gy).

### 2.3. Endpoint Toxicity Definition

The Common Terminology Criteria for Adverse Events (CTCAE) scale version 4.03 [26] was used to evaluate toxicities among patients at baseline and weekly during RT treatment. Since the definitions of dry mouth (xerostomia) had not changed from CTCAE version 3 to version 4, there was no need to convert the toxicity scores of patients treated before 2010 (evaluated using the CTCAE v. 3.0). Basic oral care was performed according to previously reported institutional guidelines [27].

The endpoint evaluated for dose-response modelling was acute salivary dysfunction grade ≥ 2, i.e., at least one grade 2 or grade 3 score at any weekly assessment throughout the RT course. According to CTAE v4.0, salivary dysfunction was scored as grade 2 when moderate symptoms were reported that required oral intake alterations (e.g., copious water, other lubricants, a diet limited to purees and/or soft, moist foods); salivary dysfunctionwas scored as a grade 3 if the inability to “adequately consume food orally” is reported or if tube feeding or total parenteral nutrition is indicated

### 2.4. Modelling and Statistical Analysis

The clinical parameters collected for this study were sex, age, smoking habit, body mass index (BMI), staging, histology, comorbidities grouped by hypertension, cardiological conditions, diabetes mellitus, haematological conditions and tumours other than NPC/HNC. Treatment variables included RT fractionation, technique (IMRT vs. volumetric modulated arc RT), overall treatment time and CHT regimens (concomitant RT-CHT with or without previous induction CHT). The schedule of platinum-based concurrent chemotherapy (3× weekly vs. weekly) and the total platinum doses administered were not considered in this analysis. The dose distribution and volume parameters of each OAR were also evaluated.

Age was split into 5-year intervals; BMI was studied both as a continuous and dichotomous variable, using 25.0 and 30.0 kg/m^2^ as the cutoff values according to the WHO definition of overweight and obese, respectively; histology was categorised as undifferentiated or squamous cell carcinoma (SCC). Overall stages and T categories were considered as ‘low-intermediate’ if the overall stage was I or II or the T category was 1, 2 or 3 and ‘high’ if the overall stage was III-IVA–IVB or the T category was 4. The N category was dichotomised as N = no (if N0 or N1) or Y = yes (if N2, N3a or N3b).

To identify a dose-response relationship for the toxicity endpoint, the equivalent uniform dose (EUD) was first investigated, as it simultaneously takes into account both the whole dose-volume histogram (DVH) and the organ architecture. The EUD was calculated from the DVH of each OAR according to the following formula [28]:

$$EUD={\left(\underset{i}{\sum }v_{i}D_{i}{}^{\frac{1}{n}}\right)}^{n}$$

where *n* varies in the range from 0.05–1 with a 0.05 step size, *n* is the volume effect factor, and *v_i_* is the volume fraction that receives the bin dose *D_i_*, (*D_i_, v_i_*) identifying a point in the differential DVH. The *n* value which best describes the association between each OAR and the endpoint was determined by EUD evaluation through a *t*-test. When this procedure did not converge, DVH cutoff points were also evaluated through a *t*-test.

Uni- and multi-variate logistic analyses were performed on the resulting dosimetric parameters and clinical- and treatment-related features to identify possible dose-response modifiers; the selection of variables was further guided by least absolute shrinkage and selection operator (LASSO). The choice of the tuning parameter for LASSO was determined by considering 5-fold cross-validation and evaluation of deviance [29].

The models, including selected dosimetric/clinical features, were based on logistic regression and evaluated through their likelihood in the training cohort. Internal validation was carried out through 1000 bootstrap resamplings and used to correct model performance measures for optimism, thus obtaining TRIPOD type 2a models [30]. The goodness of fit of the final models was evaluated with the Hosmer-Lemeshow test (with six equally sized groups) and calibration plot. Lastly, residuals were calculated to identify subpopulations of patients showing toxicity grades higher/lower than those predicted by the model, possibly due to underlying clinical and genetic dose-modifying factors without available data in this analysis.

A nomogram was drawn based on the final logistic regression model.

Following the TRIPOD statement [30], model validation in the independent cohorts was performed by calculating toxicity predictions using the original model (without any change in the constants and coefficients from the logistic fit) and these predictions were compared with the observed outcomes. Performance of the model in the external independent validation cohorts was evaluated through the area under the receiver operating a characteristic (ROC) curve (AUC) and via calibration plots (calibration in the large and calibration slope). Validation in the second NPC cohort led to a TRIPOD type 2b model (validation in a non-randomly selected population, non-random selection based on the year of treatment). In contrast, validation in the mixed non-NPC cohort led to a TRIPOD type 3 model (validation in an independent population).

## 3. Results

### 3.1. Population for Model Development

Overall, 160 NPC patients were treated at our institution between 2004 and 2015: 132 of them met the inclusion criteria (complete clinical and dosimetric data) and were therefore eligible for the study. Table 1 shows the clinical characteristics and treatment details of the development population. All patients received the treatment as planned, without breaks due to acute toxicity. The median overall treatment time was 50 days (range: 43–61 days). Volume and dose details for the selected OARs are presented in Table 2.

Throughout the RT course, acute salivary dysfunction (grade ≥ 2) was reported in 90 out of 132 patients (68.2%). The median time to the endpoint was 4 weeks and the actuarial incidence was 12% at week 2, 26.5% at week 3, 42% at week 4, 53% at week 5, 60% at week 6 and 68.2% at the end of treatment.

The best dosimetric descriptors for this endpoint were the EUD for the OC calculated with *n* = 0.05 (OC_EUD) (*p* = 0.002), i.e., a dose near the maximum OC dose, and the minimum dose for cPG (*p* = 0.01). According to ICRU recommendations, we considered the “near-minimum” dose, i.e., D98% (*p* = 0.01), for cPG in further analyses.

**Figure A1:**
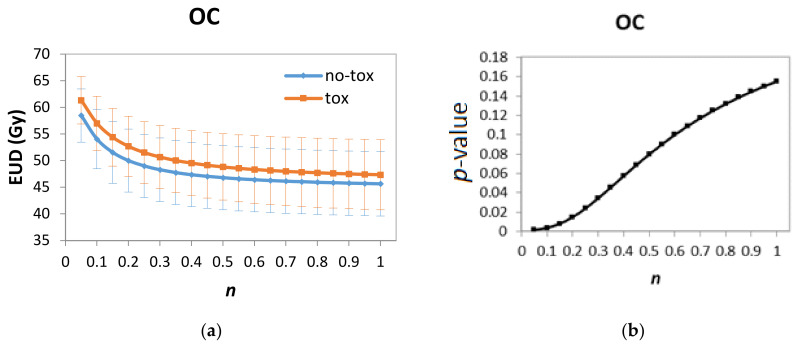
(**a**) Average Equivalent Uniform Dose (EUD) and standard deviations for oral cavity as a function of the volume parameter *n* used to calculate EUD. Results for patients with (red line, label “tox”) and without (blue line, label “no-tox”) where grade ≥ 2 acute salivary dysfunction is reported. (**b**) The *p*-values for the *t*-test comparing average EUDSs for patients with and without toxicity. The differences are statistically significant for *n* in the range 0.05–0.35.

**Figure A2:**
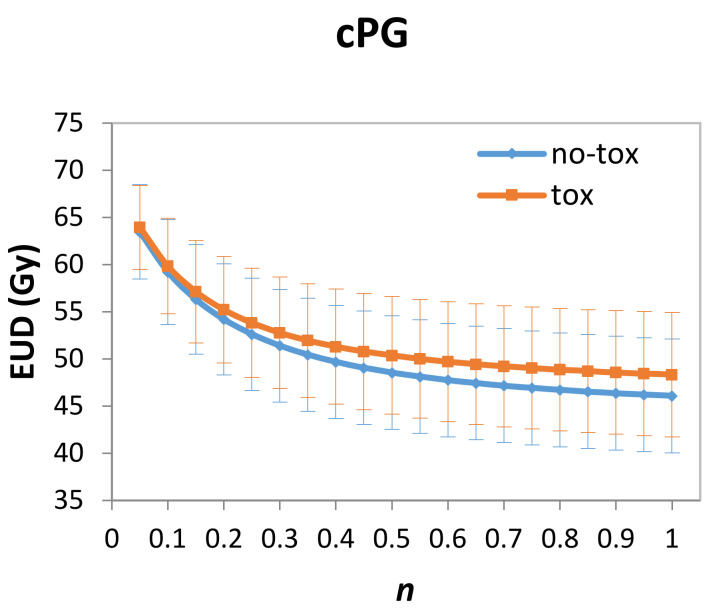
Average Equivalent Uniform Dose (EUD) and standard deviations for combined parotid glands (cPG) as a function of the volume parameter *n* used to calculate EUD. Results for patients with (red line, label “tox”) and without (blue line, label “no-tox”), where grade ≥ 2 acute salivary dysfunction are reported. There are no statistically significant differences (*t*-test) in EUD in the two subpopulations.

**Figure A3:**
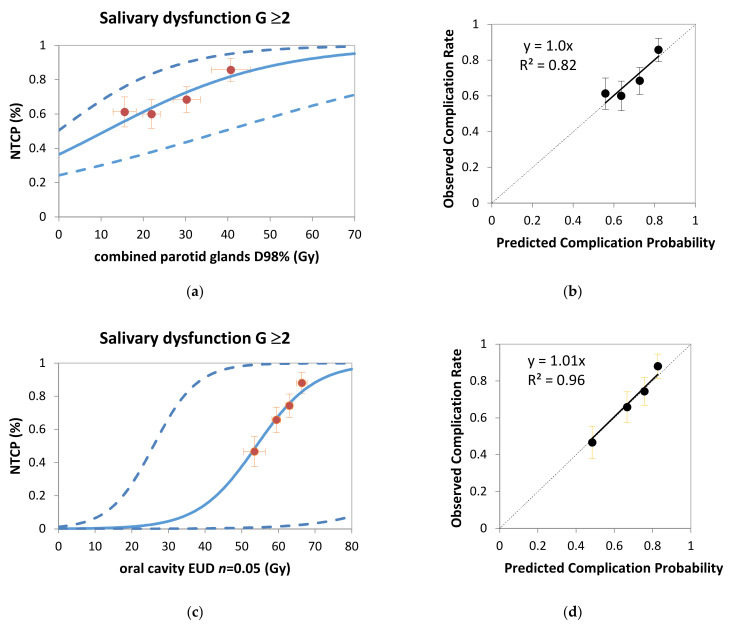
Probability of grade ≥ 2 acute salivary dysfunction as a function of D98% of combined parotid glands (panel (**a**)) and oral cavity Equivalent Uniform Dose (EUD) calculated with *n* = 0.05 (panel (**c**)). Observed complication rates (solid circles) and the predicted complication probability curve (continuous line) are plotted. The dotted lines identify the 68% confidence intervals for the predicted curve. Calibration plots for the predictive models for grade ≥ 2 acute salivary dysfunction: for the model including combined parotid glands D98% (panel (**b**)) and for the model including oral cavity EUD (panel (**d**)). The solid lines represent the calibration graphs of the models and the black points are the observed complication rates together with their standard deviation. The dashed line represents the identity line. The calibration equation together with the corresponding R^2^ are presented on the calibration plots.

In Appendix A, a dose-response curve is presented for each OAR-endpoint pair (Figure A1, Figure A2 and Figure A3), as well as the complete results of the univariable analysis of clinical and treatment-related parameters (Table A1). LASSO analysis resulted in a 4-variable model including cPG D98% (OR = 1.04, for 1 Gy increase in cPG D98%), OC_EUD (OR = 1.11, for 1 Gy increase in OC_EUD), age (OR = 1.08, for each 5-year interval increase) and smoking history (OR = 1.37, yes vs. no). Table 3 shows the model details.

**Figure 1:**
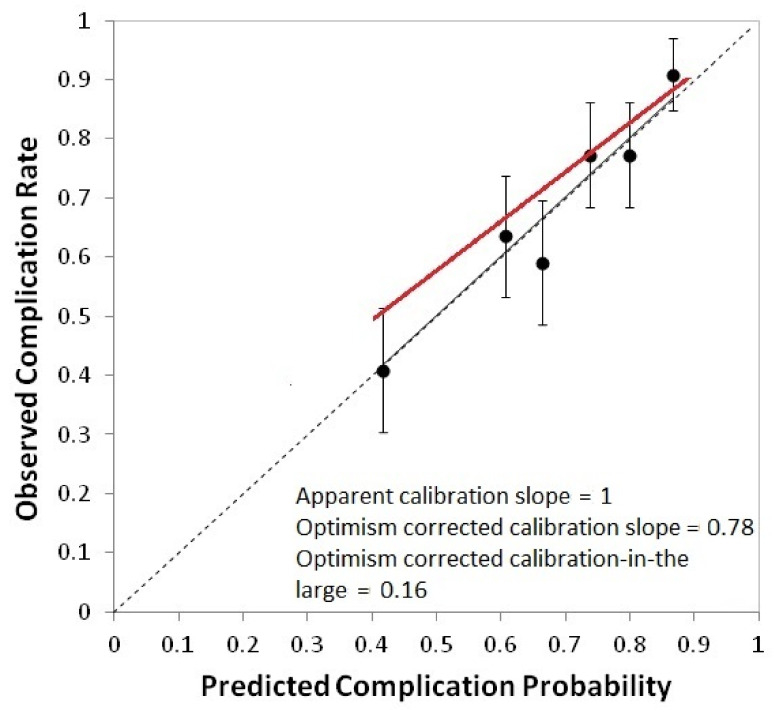
Calibration plot for the model for grade ≥ 2 acute salivary dysfunction in the nasopharyngeal cancer cohort use for model development. Calibration plots present the rate of observed events in a group of patients (*y*-axis) vs. the mean predicted probability for the same group (*x*-axis). Groups of patients are ordered for increasing predicted probability. Error bars represent the confidence interval in observed frequencies calculated from proportions in the study population and based on a normal distribution of events. The dotted line is the identity line, identifying perfect calibration, the continuous black line is the apparent calibration line, and the red line is the calibration line after correction for optimism. Calibration slope and calibration-in-the-large are also reported.

**Figure 2:**
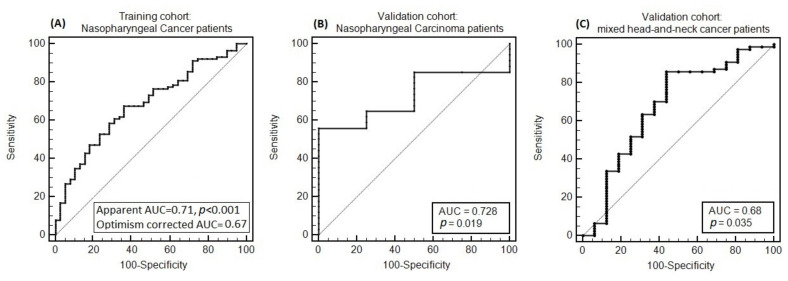
The receiver operating characteristic (ROC) curve for the model for grade ≥2 acute salivary dysfunction as applied in the three cohorts considered in this study: (**A**) nasopharyngeal cancer (NPC) cohort use for model development, (**B**) NPC validation cohort and (**C**) head and neck cancer other than NPC validation cohort (HNC).

**Figure 3:**
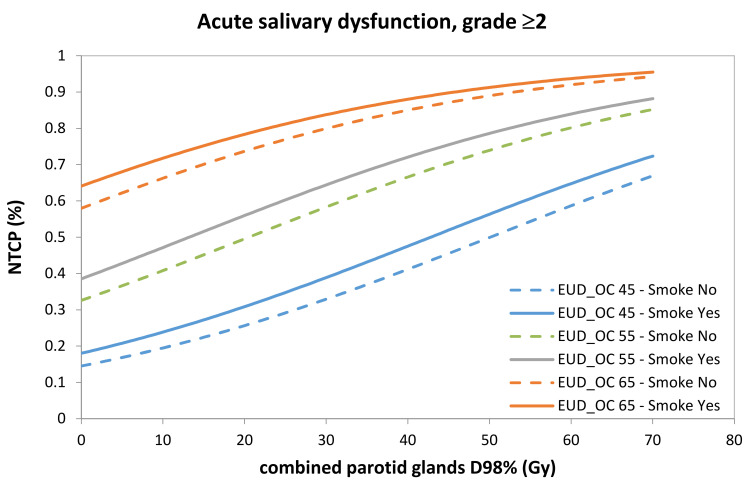
Probability of grade ≥2 acute salivary dysfunction as a function of combined parotid glands D98%. Three dose levels for the equivalent uniform dose (EUD) for oral cavity (OC) calculated with *n* = 0.05 are represented: EUD to OC = 45 Gy in blue; EUD to OC = 55 Gy in green; and EUD to OC = 65 Gy in red. Dashed and continuous curves correspond to negative or positive smoking history, respectively.

**Figure 4:**
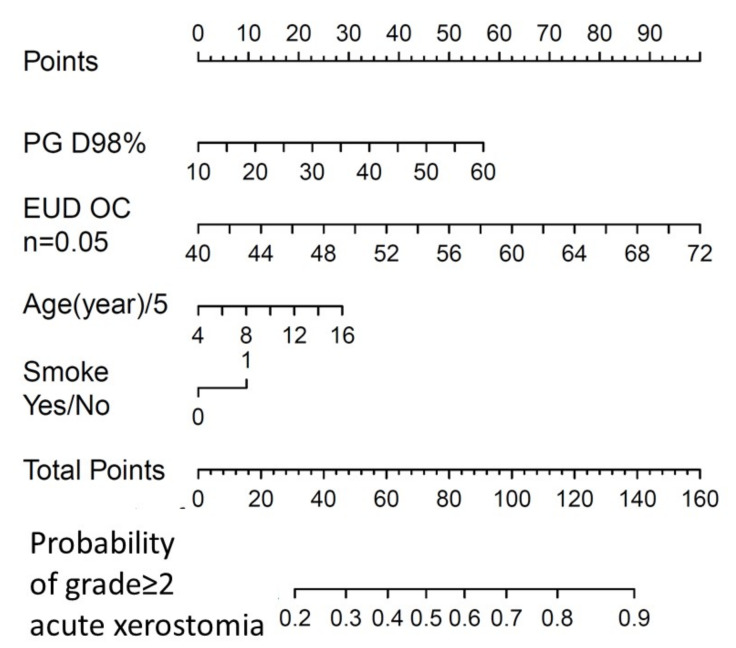
Nomogram for grade ≥2 acute salivary dysfunction as derived from the multivariable logistic regression model presented in Table 3. Age is expressed in 5-year units. cPG = combined parotid glands; OC = oral cavity; and EUD = Equivalent Uniform Dose.

Good agreement between expected and observed complication rates was implied, since the Hosmer–Lemeshow test was not significant ($\chi_{HL}^{2}=$1.2 and *p* = 0.88). A calibration plot is shown in Figure 1 (apparent calibration slope = 1.0, calibration slope after correction for optimism = 0.78, calibration-in-the-large after correction for optimism = 0.16). The apparent AUC was 0.71, while optimism-corrected AUC was 0.67; the ROC curve is plotted in Figure 2a. Normal tissue complication probability (NTPC) as a function of cPG D98% for three chosen values of OC_EUD with a smoking history categorized as ‘Yes’ or ‘No’, is presented in Figure 3, while the nomogram to predict the probability of grade ≥ 2 acute salivary dysfunction is illustrated in Figure 4.

**Figure 5:**
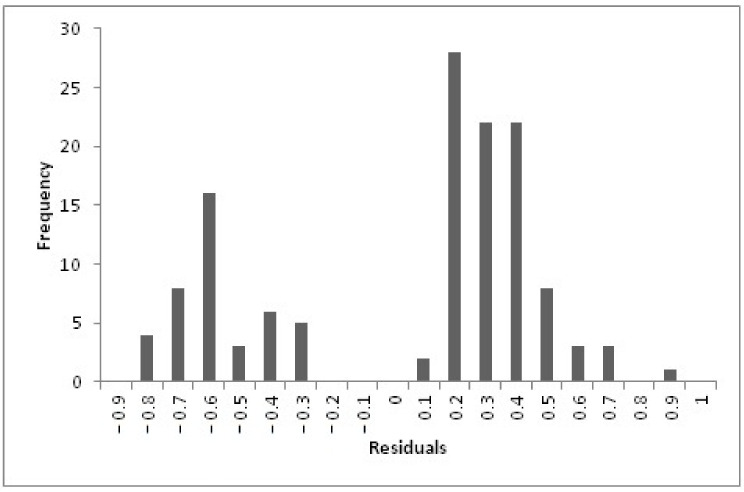
Histograms of residuals for the model for grade ≥ 2 acute salivary dysfunction.

A residuals histogram is depicted in Figure 5: only one patient had a residual ≥0.7, i.e., only one patient experienced grade ≥2 acute salivary dysfunction despite his low predicted probability. On the other hand, four patients had a residual ≤−0.7, i.e., they did not exhibit grade ≥2 acute salivary dysfunction despite their high model prediction. These patients could be classified as radioresistant patients.

### 3.2. Model Validation Population

The validation population of NPC patients (NPC_V cohort) included 38 patients, with 34 patients with grade ≥ 2 acute salivary dysfunction (89.5%); the validation population including patients with HNCs other than the NPC (HNC_V cohort) consisted of 93 patients, 77 of whom had grade ≥ 2 acute salivary dysfunction (92.8%).

As a general remark, grade ≥ 2 acute salivary dysfunction incidence was significantly different in the training and validation populations (*p* = 0.01), thus impairing calibration-in-the-large, which was 0.38 and 0.53 in the NPC_V and the HNC_V cohort, respectively. The calibration slope was promising for the NPC_V cohort (0.77), whereas the 0.50 value for HNC_V indicates a relationship less steep than expected between the observed salivary dysfunction rates and model-predicted probabilities. Age and smoking status were not confirmed as risk factors in the validation population: the model-predicted probability is thus enhanced by the presence of these factors, but this is not mirrored in the actual observed toxicity rates.

**Figure 6:**
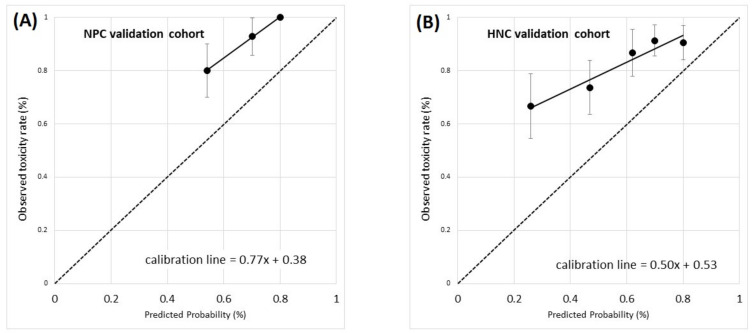
Calibration plots for validation of the model for grade ≥ 2 acute salivary dysfunction in the two independent external cohorts: (**A**) nasopharyngeal cancer (NPC) cohort and (**B**) head and neck cancer other than NPC cohort (HNC). Calibration plots present the rate of observed events in a group of patients (*y*-axis) vs. the mean predicted probability for the same group (*x*-axis). Groups of patients are ordered for increasing predicted probability. Error bars represent the confidence interval in observed frequencies calculated from proportions in the study population and based on a normal distribution of events. The dotted line is the identity line, identifying perfect calibration, the continuous black line is the calibration line. The calibration slope and calibration-in-the-large are also reported.

On the other hand, the effect size for the two dosimetric factors was confirmed: cPG_D98% with OR = 1.09 (for 1 Gy increase in cPG_D98%, 95%CI: 1.01 to 1.17) and OC_EUD with OR = 1.08 (for 1 Gy increase in OC_EUD, 95%CI: 1.02 to 1.15). The calibration plots for the two validation cohorts are presented in Figure 6A,B).

The model also confirmed good discrimination in the NPC_V cohort: the AUC was 0.73 (*p* = 0.02), and the negative predictive values (NPV) and positive predictive values (PPV) were 0.65 and 0.75 (cutoff was set at 65% of the predicted toxicity probability), respectively. The discrimination was also satisfactory in the HNC_V cohort: AUC = 0.68 (*p* = 0.035) and NPV and PPV equal to 0.52 and 0.69 (cutoff was set at 65% of the predicted toxicity probability), respectively (Figure 6).

## 4. Discussion

Acute xerostomia is one of the most frequent side effects of curative RT among HNC and NPC patients [31,32,33]. Although it does not have dose-limiting toxicity, as do mucositis and dysphagia, it can result in oral discomfort and difficulty speaking or swallowing, thus potentially contributing to a decreased nutritional intake and weight loss, substantially reducing patients’ QoL. More importantly, a link between acute salivary dysfunction and the risk of developing late dysphagia has been found, indicating a strong consequential component in this late reaction [21].

Several authors have analyzed predictive factors for xerostomia occurring within 3–6 months after treatment completion, thus an “early” form of late xerostomia [18,34,35,36], a recent review was also published [37]. To our knowledge, there are no relevant data regarding the prediction of salivary dysfunction during treatment. Therefore, our manuscript is the first to build a predictive model for this toxicity endpoint, assessing acute xerostomia according to CTCAE v. 4.0 in a homogeneous cohort with NPC-treated patients with IMRT techniques.

The present retrospective study aimed to build a predictive model for acute salivary dysfunction during curative IMRT specifically for locally advanced NPC patients. We were aware that NPC occured in a unique subsite among HNC sites. We chose this particular population because of its homogeneity for clinical characteristics and treatment strategies. In addition, we chose this population because it represented one of the most complicated subsites among head and neck cancers in terms of toxicities and radiation doses to OARs. Acute salivary dysfunction was unequivocally higher among NPC patients because all neck nodal levels were treated at least prophylactically. This translates into high doses to the major salivary glands, bilaterally.

We developed a predictive model based on two dosimetric parameters and two additional clinical factors. With regard to the dosimetric parameters, we identified cPG D98% (OR = 1.04) and EUD for the OC calculated with *n* = 0.05 (OR = 1.1) as predictors of grade ≥2 acute salivary dysfunction. Of note, both dosimetric factors were confirmed, based on their effect size, in the two populations used for independent external validation.

Much of the literature regarding xerostomia, particularly late xerostomia, focuses on high radiation doses to the contralateral PG. Many authors have underlined the importance of sparing at least the contralateral gland to not compromise salivary flow after RT [34,38]. Conversely, according to the clinical and dosimetric characteristics of our training population, notably the impossibility of discerning between ipsilateral and contralateral PGs, we did not obtain a similar finding.

Our first result regarding the role of cPG D98% (*p* = 0.01) is quite surprising and difficult to explain. This finding may suggest a uniform biological response of the whole glandular volume to medium-high doses of radiation sufficient to result in moderate (grade ≥ 2) acute xerostomia, when combined with a non-negligible dose to the oral cavity. However, van Luijk et al. found that some parts of PGs were more sensitive to radiation than others, i.e., the regions containing the major ducts where the stem and progenitor cells resided. The doses to these parts of the glands were consistent predictors of salivary dysfunction 1 year post-RT in 74 HNC patients [39]. Very recently, Jiang et al. also identified specific subvolumes in parotid and submandibular glands predictive of xerostomia 3 months after treatment (in 195 HNC patients) by applying machine learning methods to voxel dosimetry [40].

Regardless of the actual explanation, our findings suggested, firstly, that D98% could be considered a parameter associated with salivary dysfunction during treatment and, secondly, that more attention should be paid to the minimum dose applied to PGs during plan optimization, especially when the mean doses are high and PGs are largely involved in the irradiated volume, as is the case in NPC treatments where PGs received at least one prophylactic dose.

In contrast to the most common findings in the literature, we did not find cPG mean doses as predictive of severe xerostomia during RT. However, different dose parameters were often strongly correlated (the Pearson correlation coefficient for cPG D98% and the mean dose was 0.88 in our study, with *p*-value < 0.001). Until a few years ago, the prediction of late salivary alterations was generally based on the PG mean dose only. More specifically, the QUANTEC (Quantitative Analyses of Normal Tissue Effects in the Clinic) reported that severe xerostomia, defined as long-term stimulated salivary flow of <25% of the baseline, can be reduced if at least one PG (or both) is spared with a mean dose lower than 20 or 25 Gy, respectively [15,16]. However, it is clear that the assessment of physician- and patient-rated xerostomia is much more complex and does not necessarily deal with only one OAR [24]. Jellema et al. found that patient-rated xerostomia, 6 months after curative RT, and assessed according to the EORTC QLQ-H&N35 questionnaire in 113 HNC patients, significantly depended on the mean dose to both the parotid and submandibular glands [41]. Lee et al. [42] also proposed a model for the prediction of xerostomia at 1 month after the end of RT and included the mean dose to the ipsilateral submandibular gland, the mean dose to the contralateral submandibular gland, and the mean dose to the oral cavity (in a population of 67 patients with NPC). Of note, we could not include submandibular glands in the development of our model because their inclusion in PTVs was reflected by their mean doses approximately equal to the PTV prescription dose, which is beyond the known supposed dose constraint for their sparing [15]. This happened because the majority of NPC patients in our cohort had N2-N3 categories with bulky and radiological extracapsular extension of IIA levels. In those cases, according to our internal policy, the ipsilateral IB level was included in the intermediate- or low-risk target volume. Another condition of including IB level in the target volume was lymph nodal positivity at that level. Notably, in the training of the NPC cohort, 128 patients (97%) had pathological lymph nodes. Furthermore, 118 patients (89.4%) met at least one of the three criteria mentioned above. There were 33 patients (86.8%) with positive nodes and 29 patients (87.9%) with at least one of the reported criteria in the validation NPC cohort. Being that the dose to the submandibular glands was almost homogeneous in this population, it cannot result in an association with the considered endpoint. This does not mean that the dose to the submandibular glands is of no importance, but instead that in this kind of treatment, where this dose is unavoidably higher, other organs play a role, and we could try to spare these organs to reduce the risk of mouth dryness. The importance of the submandibular glands for the basal level of saliva production is indirectly witnessed in our population by the high rate of acute xerostomia after the high dose to these glands. Still, the modulation of these doses cannot be applied due to the clinical setting or be considered as a radiotherapy treatment optimisation goal.

Since xerostomia is multifactorial, clinical parameters should also be added to increase the predictive performance of NTCP models [17,35,42,43]. Beetz et al. [35] demonstrated that the mean dose administered to PGs failed to identify all patients at risk of late xerostomia (assessed according to EORTC QLQ-H&N35) among the 307 HNC cases. In particular, implementing the QUANTEC criteria was not sufficient to prevent persistent, moderate-to-severe patient-rated xerostomia in elderly patients (age > 70 years old) and patients already complaining of minor xerostomia at the baseline [44].

More recently, Gabryś et al. demonstrated that, in highly conformal RT treatments, the prediction of xerostomia, rated according to the CTCAE scale in 153 HNC patients, could be enhanced by incorporating organ- and dose-shape descriptors (i.e., parotid volume, eccentricity and DVH spread) [45]. They also remarked that future NTCP models for xerostomia would need to generate personalised data-driven risk profiles, as their data still strongly relied on patient-specific, dose-independent factors, such as parotid shape or patient sex. However, models developed based on real-world data are a valuable complement to clinical trials and are particularly useful as decision-support tools in a learning healthcare system. A registry study of the modelling of xerostomia after RT in HNC patients from Karolinska University reported that the role of the parotid dose may be of lower importance compared to clinical variables in a heterogeneous population [46].

A second significant parameter was EUD for OC, calculated with *n* = 0.05 (OR = 1.11, *p* = 0.002), i.e., nearly a maximum dose to the OC. The very low *n*-value for the OC highlighted a somewhat serial behaviour of the organ for this kind of injury. This means that even small volumes receiving high doses were significantly associated with the toxicity. This particular result could be related to the inclusion of other glands, especially the minor salivary glands (those located in the most posterior part of the volume overlapping the PTVs), in OC delineation, as the OC was contoured according to Eisbruch [25]. If this portion of the OC receives a full curative radiation dose, it could impact the development of acute xerostomia. Interestingly, in our previous report on the prediction of severe acute oral mucositis, the EUD for the OC with *n* = 0.05 was included in a 3-variable model (together with a PG mean dose and BMI). This underlined the strict interdependence between acute oral mucositis and xerostomia [47].

The role of minor salivary glands has been more exhaustively investigated in connection with late xerostomia. In the PARSPORT study [38], the authors reported a slightly discordant relationship between measurable salivary flow and grade ≥ 2 late xerostomia, likely because of a different xerostomia perception by the patients and the contribution of other factors, such as radiation-induced damage to the oral mucosa and other salivary glands (minor salivary glands, submandibular glands). Eisbruch et al. [25] found a significant relationship between the mean dose to the OC (representing the dose received by the minor salivary glands) and patient-rated late xerostomia in a series of 132 HNC patients treated with curative IMRT. In contrast, other authors did not find a significant association [35,41], possibly due to differences in the OC volume delineation among various studies or in the tools employed to assess xerostomia.

As mentioned before, radiation-induced late xerostomia relates to more than just the damage to PGs; tumour stage, age, and smoking habit [17,35,36,43] could also influence its development. Our study found that older age and smoking history also had a predictive role for grade ≥ 2 acute xerostomia (OR = 1.08 and =1.37, respectively). Elderly patients suffered from salivary dysfunction more than younger patients because salivary flow physiologically decreases with age, and so elderly patients are more likely to have comorbidities and use medications that may affect and reduce saliva production at rest [34,42,48]. Of note, it was not possible to include the specific use of xerogenic drugs, as the size of the population coupled to the large number of drugs causing mouth dryness led to sparse results. We tried to use older age as a surrogate of the high frequency of polypharmacy and to correct the possible bias in results, taking the baseline presence of mouth dryness into account.

Among individual habits, smoking was recognised as a contributor to xerostomia after IMRT for HNC because it reduced the serous component of salivary flow [42]. Unlike age, smoking had a smaller effect size, which might be due to the low prevalence of smoking in our cohort (18%). Nonetheless, we decided to include it in the model to better tailor it to patients’ supportive care during treatment.

These two clinical risk factors (older age and smoking habit) were not confirmed in our two validation cohorts, indicating that larger, more prospectively followed populations were needed to obtain clear information on the possible roles of these factors as modifiers of the dose-response curve.

Of note ECOG (Eastern Cooperative Oncology Group) or the WHO performance statuses were not included in the statistical analysis due to the retrospective nature of the training NPC cohort. However, we considered in the study the major clinical factors known to impact xerostomia, e.g., use of chemotherapy, comorbidities and age.

External independent model validation was carried out in two different cohorts: (a) one NPC cohort, similar to the one used for model development, and (b) one mixed HNC cohort. This second cohort was chosen to investigate the generalizability of the predictive model, acknowledging the clinical need of such a model even outside NPC subsites, with a high percentage of other HNC patients presented with the difficulty of sparing contralateral parotid and/or submandibular glands because of the laterality of the disease. The proposed model could then be inserted in the frame of TRIPOD type 2b (model development and validation in a population derived from non-random splitting; the NPC cohort, with non-random splitting based on the treatment year) and of TRIPOD type 3 models (model development and validation made by the same researchers but on separated populations) [30].

**Figure A4:**
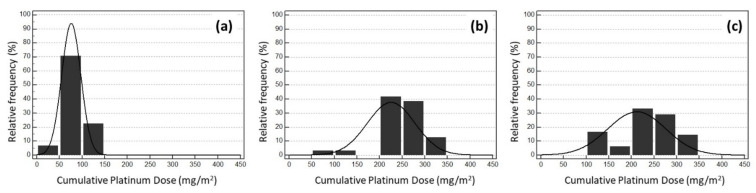
Distribution of cumulative platinum doses in the development cohort (panel (**a**)) and in the two validation cohorts (panels (**b**,**c**)).

The first remarkable finding in the validation cohorts was a large significant increase in acute salivary dysfunction rates from 68% to ~90% (*p* < 0.001). This type of miscalibration (reflected in the calibration-in-the-large) is a typical finding in external validation studies. It indicates that some patient’s characteristics, which were not embedded in the prediction model, were distributed in a different way in the validation sample compared with the sample used for the development. In this study, the main difference between the development cohort and the validation cohorts was in the historical period (2004–2015 for the development cohort and 2017–2018 for the validation cohorts). In this time frame, the main changes in chemo-radiation treatment included more intensive use of accelerated radiotherapy with the increased use of a simultaneous boost at 2.12–2.22 Gy (23.5% in patients with a simultaneous boost at 2.12 Gy/day in the development of the NPC cohort, vs. 97.4% in the validation of the NPC cohort, vs. 65.6% in the mixed head and neck validation cohort), and increased cumulative doses of platinum-based chemotherapy (distribution of cumulative doses in the three population is reported in Figure A4 in the Appendix A; median cumulative doses were 75 mg/m^2^ in the NPC development cohort, vs. 250 mg/m^2^ in the validation NPC cohort, vs. 200 mg/m^2^ in the mixed head and neck validation cohort). Of note, the rate of grade ≥ 2 acute xerostomia in the mixed head and neck validation cohort was higher in patients treated with simultaneous boosts at ≥2.12 Gy (85% vs. 78%, although the difference was not statistically significant). In the NPC validation population, simultaneous boosts at ≥2.12 Gy were used in all patients but one, so a comparison of toxicity rates cannot be performed. A further possible difference between the development and validation populations resided in the purpose of data collection. In all cases, toxicity was scored once a week using the same CTCAE scoring system. However, for the development cohort, the assessment was done in the routine clinical context, and information was retrieved later from patients’ chart. On the other hand, the two validation cohorts were enrolled in a prospective trial explicitly designed with the aim of modelling radio-induced toxicity, and the information was directly and prospectively retrieved from the study case report forms. In both cases, the weekly information was complete. Yet, defining the endpoint as peak toxicity, with even just one grade 2 or grade 3 event determining the endpoint occurrence, could lead to significant differences in the observed toxicity rates. Careful evaluation of the time patterns of acute salivary dysfunction is in progress. Additionally, it should be determined whether alternative definitions of the toxicity endpoint could be of more significant clinical and modelling relevance, considering, for example, a longitudinal definition of toxicity, like the one used for acute oral mucositis modelling in previously published studies [47,49,50]. Notably, when considering a longitudinal definition, as the mean grade of xerostomia during treatment, more stable results were produced, with 25% of patients having a mean grade of ≥1.5 in the NPC development cohort, 36% (*p* = 0.15) and 29% (*p* = 0.50) in the NPC validation and mixed head and neck validation populations, respectively.

Despite the aforementioned differences in the toxicity rates, which leads to an impaired calibration-in-the-large for the validation populations, the effect sizes of the two dosimetric factors included in the model was confirmed (cPG_D98% with OR = 1.09 for 1 Gy increase in cPG_D98% and OC_EUD with OR = 1.08 for 1 Gy increase in OC_EUD) together with AUC (0.67 in the development population and 0.73/0.68 in the validation populations). On the other hand, clinical features (age and smoking status) were not confirmed as risk factors.

A limitation of our study was the retrospective nature of the analysis. This poses some issues on the possible inter-clinician variability in the weekly scoring of toxicity, possibly accompanied by different underlining factors and not an “explicit” shift in scoring with time (the training population was treated in 2004–2015). Validation populations were enrolled prospectively in a limited timeframe (18 months) and within a study explicitly devoted to the modelling of radio-induced toxicity, thus involving two radiation oncologists who weekly scored all patients.

A second limitation was the small number of patients in both the training cohort and in the validation cohort compared to regions where NPC was endemic. Yet, this was one of the major mono-institutional series of consecutive NPC patients managed with IMRT techniques in low incidence geographic areas.

A third limitation was a lack of some more “modern” features that could have helped to identify variability at the single-patient level, such as genetic features, inflammatory markers, radiomic features [19,20,45,51] or delta-radiomics [52,53,54,55]. Inclusion of this information increased the performance of predictive models for the prediction of either early xerostomia (during treatment or at 3–6 months after RT completion) or late/chronic toxicity (>6 months after RT completion). Notably, the delta-radiomics approach (based on CTs used for CT-guided RT) was also successfully adopted for the prediction of xerostomia at the end of treatment in an analysis of small populations (35 NPC patients in [52] and 59 patients in [55]). The two studies focused on changes in CT images occurring at very different times during treatment, the second week vs. the fifth week, yet both suggested a possible guide for an adaptive treatment in the following weeks.

A further possible limitation is the use of the DVH as summary dose metrics. Some recent approaches proposed the analysis of doses at the voxel level [40], thus allowing the possibility of highlighting more specific local patterns in the dose distribution. This approach seems promising in the whole field of normal tissue dose-response modelling, yet it is still in a phase of hypothesis-generating studies, instead of being at the stage of possible implementation in treatment optimisation. For these reasons, we chose to start with a classical DVH-based NTCP approach. Further analysis is in progress.

A strength of this study is that it relied on the use of detailed dosimetric information with the evaluation of a possible interaction between different OARs in terms of dose/volume parameters and that the model results were tested in two independent populations. Model testing was performed without any adjustment of parameters. As we were aware that our model was developed in a highly homogeneous population in terms of disease site (NPC only), we chose to test it both in a similar population of NPC patients and in a different, more general, population of mixed HNC patients, thus determining whether this model could be applied in various clinical situations. Validation results highlighted that the dosimetric part of the predictive model is highly generalisable, whereas the clinical risk part remains a weak component. The validation confirmation of the discriminative power of the model and of the effect size of the dosimetric variables motivate confidence in the factors to be optimised while planning RT.

These types of models, including external validation (TRIPOD types 2b and type 3), are still largely lacking. The review by Sharabiani and colleagues [37] highlighted that out of 18 identified models for xerostomia, only one model was type 2b and one was type 3, with two type 4 models (validation in an external population made by independent researchers).

## 5. Conclusions

We built a predictive model for acute salivary dysfunction among NPC patients receiving combined treatment. Our results showed that a near-minimum dose (D98%) to the cPG and high doses to the small OC volumes were associated with grade ≥2 acute xerostomia, with older age and smoking history acting as dose-modifying factors. The model was tested in two independent external cohorts, with the findings confirmed for the dosimetric component. In contrast, the contribution of clinical risk factors was not confirmed and still requires further evaluation. We aim to improve patients’ QoL and care by detecting those at a higher risk of xerostomia development during treatment, being aware that late xerostomia could develop from acute xerostomia. Of note, data from prospective trials in our institution highlight an association between late grade ≥2 xerostomia and acute grade ≥2 salivary dysfunction; *p* = 0.04 for the Chi-squared test and OR = 11. Another implication, from a planning point of view, is the possibility of paying particular attention to the identified dosimetric parameters. Further variables, e.g., gene-, radiomic- or microenvironment-related, should be investigated to possibly explain the radiosensitivity/radio resistance exhibited by some patients and tailor better clinical care to patients’ needs as result.

## Figures and Tables

**Table 1:** Patient and treatment characteristics for the three cohorts considered in the study: (a) Nasopharyngeal Cancer (NCP) patients for model development; (b) NPC patients for model validation and (c) mixed head and neck cancer (other than NPC) validation population.

Patient Characteristics		(a) NPC Development (*n* = 132 pts) (%)	(b) NPC Validation (*n* = 38 pts) (%)	(c) HNC Validation (*n* = 93 pts) (%)
Gender	Female	40 (30.3)	8 (21.1)	25 (26.9)
	Male	92 (69.7)	30 (78.9)	68 (73.1)
Age median {range} (years)		49 {18–81}	52 {24–72}	62 {23–83}
BMI median {range} kg/m^2^		25.9 {16.6–42.9}	25.8 {18.2–32.9}	24.8 {14.7–36.4}
Smoke (Yes)		24 (18.2)	20 (52.6)	63 (67.7)
Comorbidities (Yes)	Hypertension	19 (14.4)	10 (26.3)	37 (39.8)
	Diabetes mellitus	4 (3.0)	1 (2.6)	4 (4.3)
	Cardiological	6 (4.5)	3 (7.9)	20 (21.5)
	Haematological	7 (5.3)	-	2 (2.1)
	Oncological	5 (3.8)	3 (7.9)	15 (16.1)
Histology (WHO tumour classification)	Undifferentiated	119 (90.2)	34 (89.5)	-
	SCC	13 (9.8)	4 (10.5)	84 (90.3)
	Other		-	9 (9.7)
Staging procedures	MRI	129 (97.7)	38 (100)	86 (92.5)
	(^18^F)FDG-PET	123 (93.2)	38 (100)	88 (94.6)
Stage (Edge 2010)	II	20 (15.1)	6 (15.8)	7 (7.5)
	III	38 (28.8)	12 (31.6)	28 (30.1)
	IVA	29 (22.0)	9 (23.7)	45 (48.4)
	IVB	45 (34.1)	11 (28.9)	13 (14)
Treatment	RT-CHT	30 (22.7)	19 (50.0)	56 (60.2)
	iCHT + RT-CHT	102 (77.3)	16 (42.1)	11 (11.8)
	RT alone		3 (7.9)	26 (28.0)
RT technique	IMRT	70 (53.0)	-	-
	VMAT	62 (47.0)	38 (100)	93 (100)
Fractionation	2 Gy/fraction	101 (76.5)	1 (2.6)	32 (34.4)
	≥2.12 Gy/fraction	31 (23.5)	37 (97.4)	61 (65.6)

Abbreviations: NPC = nasopharyngeal cancer; HNC = head and neck cancer; pts = patients; BMI = body mass index; SCC = squamous cell carcinoma; RT-CHT = platinum-based chemotherapy concomitant to RT; iCHT = induction chemotherapy: TPF—docetaxel 75 mg/m^2^ and cisplatin 75 mg/m^2^ on day 1, and 5-FU 750 mg/m^2^/day (96-h continuous infusion)-scheme; IMRT = intensity modulated radiotherapy; and VMAT = volumetric modulated arc radiotherapy.

**Table 2:** Volumes of organs at risk and the distribution of dosimetric parameters for the Nasopharyngeal Cancer cohort used for model development.

Organ at Risk	Variable	Median	Mean ± Standard Deviation
Combined parotid glands (cPG)	Volume (cc)	51.3	53.3 ± 19.3
	Mean dose (Gy)	47.5	47.2 ± 9.5
	Maximum dose (Gy)	74.0	74.2 ± 3.5
	D98% (Gy)	25.5	26.8 ± 9.5
Oral cavity (OC)	Volume (cc)	63.3	64.8 ± 20.4
	Mean dose (Gy)	45.9	46.7 ± 6.4
	Maximum dose (Gy)	72.6	71.9 ± 3.7

**Table 3:** Multivariate logistic model for grade ≥ 2 acute salivary dysfunction.

Variable	Coeff	Standard Error	Odds Ratio	95% Confidence Interval for OR
Acute salivary dysfunction, Grade ≥ 2				
Combined parotid glands D98% (Gy)	0.038	0.023	1.04	0.99–1.09
Oral cavity EUD (*n* = 0.05) (Gy)	0.103	0.043	1.11	1.02–1.21
Age (5-year interval)	0.079	0.074	1.08	0.94–1.25
Smoking history (Yes)	0.318	0.540	1.37	0.48–3.96
*Constant*	*−7.233*	*2.620*		

EUD = equivalent uniform dose; OR = Odds Ratio; coeff = beta-coefficient.

## Data Availability

The data presented in this study are available on request from the corresponding author.

## Appendix A

**Table A1:** Results of the univariate logistic regression analysis with acute salivary dysfunction grade ≥ 2 as primary endpoint. Age was divided in five year intervals; Body Mass Index (BMI) was considered as a continuous variable and as a dichotomous one, with cutoff values set at 25.0 kg/m^2^ and 30.0 kg/m^2^ according to WHO definition of overweight and obese, respectively; histology was grouped into undifferentiated versus Squamous Cell Carcinoma (SCC); stage was dichotomised in ‘low-intermediate’ if I, II, III and ‘high’ if IVA or IVB; T stage was considered as ‘low-intermediate’ if 1, 2, 3 and ‘high’ if 4. Nodal status is 0 if N0 or N1, 1 if N2, N3a or N3b.

Variable	Coefficient	SE	*p*-Value	OR	95% CI
Gender = Male vs. Female	−0.470	0.426	0.27	0.63	0.27–1.44
Age (5-year interval)	0.064	0.070	0.36	1.07	0.93–1.22
BMI (continuous variable)	0.023	0.041	0.57	1.02	0.94–1.11
BMI ≥ 25 kg/m^2^ = 1	0.066	0.382	0.86	1.07	0.51–2.26
BMI ≥ 30 kg/m^2^ = 1	0.223	0.491	0.65	1.25	0.48–3.27
Smoke = Yes	0.405	0.514	0.43	1.50	0.55–4.11
Comorbidities					
Hypertension = yes	0.013	0.533	0.98	1.01	0.36–2.88
Diabetes mellitus = yes	5.483	6.752	0.42	240.57	0.0–1.3 × 10^8^
Cardiological = yes	0.880	1.112	0.43	2.41	0.27–21.32
Haematological = yes	1.075	1.097	0.33	2.93	0.34–25.13
Histology = SCC (reference) vs. undifferentiated	−0.054	0.632	0.93	0.95	0.27–3.27
Stage low-intermediate (reference) vs. high	0.065	0.377	0.86	1.07	0.51–2.23
T low-intermediate (reference) vs. high	−0.606	0.438	0.17	0.55	0.23–1.29
N 0 (reference) vs. 1	0.019	0.456	0.97	1.02	0.42–2.49
GTVN Volume (cc)	0.024	0.026	0.36	1.02	0.97–1.08
Chemotherapy RT-CHT (reference) vs. iCHT + RT-CHT	0.090	0.442	0.84	1.09	0.46–2.60
RT Mode IMRT (reference) vs. VMAT	0.243	0.376	0.52	1.28	0.61–2.67
Fractionation 2.0 Gy/fraction (reference) vs 2.12 Gy/fraction	−0.217	0.433	0.62	0.81	0.34–1.88
DOSIMETRIC PARAMETERS					
cPG D98% (continuous)	0.051	0.022	0.02	1.05	1.01–1.10
*Constant*	−*0.557*	*0.577*			
OC EUD (n = 0.05, continuous)	0.126	0.041	0.002	1.13	1.05–1.23
*Constant*	−*6.809*	*2.474*			

Abbreviations: SE = standard error; OR = odds ratio; CI = confidence interval; GTVN = nodal gross target volume, OC = oral cavity, cPG = combined parotid glands, EUD = equivalent uniform dose, RT = radiotherapy, RT-CHT = concomitant chemoradiotherapy; iCHT = induction chemotherapy; IMRT = intensity.
